# PLK1 protects against sepsis-induced intestinal barrier dysfunction

**DOI:** 10.1038/s41598-018-19573-x

**Published:** 2018-01-18

**Authors:** Yingya Cao, Qun Chen, Zhen Wang, Tao Yu, Jingyi Wu, Xiaogan Jiang, Xiaoju Jin, Weihua Lu

**Affiliations:** Department of Intensive Care Unit, Yijishan Hospital, Wannan Medical College, Wuhu, 241001 Anhui China

## Abstract

Sepsis and sepsis-associated intestinal barrier dysfunction are common in intensive care units, with high mortality. The aim of this study is to investigate whether Polo-like kinase 1 (PLK1) ameliorates sepsis-induced intestinal barrier dysfunction in the intestinal epithelium. The mouse intestinal barrier was disrupted after Lipopolysaccharide (LPS) injection due to intestinal epithelial cell apoptosis and proliferation inhibition, accompanied by decreased PLK1. In HT-29 intestinal epithelial cells, LPS stimulation induced cell apoptosis and inhibited cell proliferation. Overexpression of PLK1 partly rescued the apoptosis and proliferation inhibition in HT29 cells caused by LPS. Finally, LPS stimulation promoted the reduction of PLK1, resulting in apoptosis and proliferation inhibition in intestinal epithelial cells, disrupting the intestinal epithelial barrier. These findings indicate that PLK1 might be a potential therapeutic target for the treatment of sepsis-induced intestinal barrier dysfunction.

## Introduction

Sepsis is defined as a life-threatening organ dysfunction caused by a dysregulated host response to infection, which may lead to tissue and organ injures and finally to death^1^. Despite advances in management, sepsis remains the dominant challenge in the care of critically ill patients for its unacceptable morbidity and mortality rates^2^.

The intestine, which is very vulnerable to the effects of sepsis, plays a crucial role in the pathophysiology of sepsis. Indeed, it has been defined as the motor of sepsis^3^. The intestinal barrier prevents the entry of bacteria and toxins into the circulation^4^. During sepsis, the barrier is disrupted, provide an outlet for viable bacteria and their antigens to move to other locations, leading to the development or aggravation of sepsis^5^. Hence, maintenance of the intestinal barrier is critical for sepsis prevention and treatment.

The main component of the mucosal barrier is the intestinal epithelium, which mostly consists of epithelial cells. Some pro-inflammatory cytokines, such as TNF-α, can induce apoptosis of epithelial cells and thereby disrupt intestinal epithelial barrier function^6,7^. Apoptosis is a form of programmed cell death, and inhibition of sepsis-induced intestinal apoptosis increases survival rates in sepsis, although the underlying mechanisms are unknown^8^.

PLK1 is a highly conserved serine (Ser)/threonine (Thr) kinase that has been implicated in the control of cell-cycle progression and mitosis and regulates a multitude of mitotic processes^9^. Knockdown of PLK1 induces mitotic arrest and apoptosis in several human cancer cell lines^10,11^. The stability of the intestinal mucosal barrier depends on the balance of proliferation and apoptosis of intestinal epithelial cells.

The role of sepsis-induced intestinal mucosal barrier dysfunction has not been extensively studied. In this study, we assessed apoptosis and proliferation in intestinal mucosal cells in sepsis and detected the expression of PLK1. PLK1 may be a novel player in the underlying molecular mechanism of sepsis-induced intestinal barrier dysfunction.

## Materials and Methods

### Animals and sepsis model

This study was approved by the Ethics Committee/Institutional Review Board of Wannan Medical College Yijishan Hospital. All animals were treated in accordance with the guidelines of the NIH’s Guide for the Care and Use of Laboratory Animals and followed the guidelines of the International Association for the Study of Pain (IASP). Twenty C57/BL male mice (10–12 weeks, 20–25 g), purchased from HFK Bioscience, Beijing, China, were randomized and assigned to two equal groups. The LPS groups were injected intraperitoneally with 20 mg/kg LPS (Sigma 055:B5, L2880) to establish the sepsis models. The control groups were injected with an equivalent amount of normal saline.

### Sample collection and handling

Twelve hours after injection with LPS or saline, mice were killed and blood samples were collected. The blood samples were centrifuged at 3000 g for 15 min at 4 °C, and the serum was separated from clotted blood and stored at −80 °C for use in assays. Intestinal tissue samples were collected for histopathologic examination, immunohistochemistry, and western blotting.

### Enzyme-linked immunosorbent assay (ELISA)

To measure the diamine oxidase (DAO) in serum, the serum samples were thawed at 37 °C for 1 h, and DAO was detected with an ELISA kit (Mlbio, Shanghai, China), according to the manufacturer’s instructions. The experiment was repeated three times, and the results are presented as the mean value.

### Histopathology and immunohistochemistry

Intestinal tissues were fixed in 10% neutral buffered formalin, transferred to phosphate-buffered saline (PBS; pH 7.4), and sectioned (4 mm thick). Then, some of the sections were stained with hematoxylin and eosin (H&E) and the others was prepared for immunohistochemical (IHC) analysis as described^12^. Accordingly, the slides were deparaffinized, rehydrated, and immersed in 3% hydrogen peroxide solution for 10 min. Antigen retrieval was performed by heating samples in citrate buffer at 95 °C for 25 min and cooled at room temperature for 60 min. After each incubation step, the slides were washed with PBS (pH 7.4). Then, the slides were incubated separately with anti-PLK1 antibody (dilution 1:500, Abcam, England) and anti-Ki67 antibody (dilution 1:500, Cell Signaling Technology) overnight at 4 °C. Immunostaining was performed by the use of the PV-9000 Polymer Detection System with diaminobenzidine according to the manufacturer’s recommendations (GBI Labs). Slides were subsequently counterstained with haematoxylin.

### Intestinal epithelial apoptosis

Apoptotic cells in intestinal epithelium were detected with the terminal deoxynucleotidyl transferase-mediated deoxyuridinetriphosphate nick-end labeling (TUNEL) assay, by use of the DeadEnd TM Fluorometric TUNEL system (Promega, Madison, WI) on deparaffinized and rehydrated tissue sections, according to the manufacturer’s protocol.

### Cell culture and treatment

The human colorectal cancer cell line HT-29 was purchased from Basic Medical College of Peking Union Medical College, Beijing. The cells were cultured in RPMI 1640 medium supplemented with 10% foetal bovine serum (Invitrogen, San Diego, CA), penicillin (100 U/mL), and streptomycin (100 mg/mL) at 37 °C under 5% CO _2_ in a humidified incubator. The HT-29 cells were incubated with LPS at various concentrations and times. Vehicle-treated cells were used as controls. The cells were then harvested for ensuing tests.

### Plasmid construction and transfection

The full-length human PLK1 coding region was amplified from total cDNA with forward primer 5′-CCGCTCGAGGGAGATGAGTGCTGCAGTGAC-3′ with an XhoI site and the reverse primer 5′-CCGGAATTCCTATTAGGAGGCCTTGAGACGG-3′ with an EcoRI site. The amplified sequence was inserted into pcDNA 3.1 to generate pcDNA-PLK1-myc. The construction was confirmed with DNA sequencing. HT-29 cells were then transfected with the plasmid vectors by use of Lipofectamine 2000 (Invitrogen, San Diego, CA).

### Apoptosis detection

Apoptotic cells were double-labelled with AnnexinV–fluorescein isothiocyanate and propidium iodide using the Annexin V/FITC kit (Neo Bioscience, Beijing, China) and analysed with a BD^TM^ LSRΙΙ flow cytometer (BD Biosciences). Annexin V-positive cells were counted and defined as apoptotic cells. The experiment was repeated three times, and the results are presented as the mean value.

### Cell survival assays

The effects of LPS on viability of HT29 cells were assessed with a Cell Counting Kit-8 (CCK-8, Dojindo, Japan). Briefly, the cells were plated in 96-well plates. After treatment with plumbagin at the indicated concentrations and times, CCK-8 (10 μl) was added to each well and incubated at 37 °C for 1 h. The absorbance (450 nm) was measured using a microplate spectrophotometer.

### Western blot assays

Western blotting was used to determine the levels of cellular proteins. Cells were washed with cold PBS and then lysed in a radioimmunoprecipitation assay lysis buffer containing protease inhibitor and phosphatase inhibitor cocktails. The total protein concentrations were measured using the Protein Assay Kit (Bio-Rad, Richmond, CA). Equal amounts of protein samples (30–80 μg) were electrophoresed by 10% sodium dodecyl sulfate–polyacrylamide gel electrophoresis (SDS-PAGE), and then the resolved proteins were transferred to polyvinylidene fluoride (PVDF) membranes (Millipore, Bedford, MA, USA) at 100 V for 1 hour at 4 °C. Subsequently, membranes were blocked with Tris Buffered Saline Tween (TBST) containing 5% non-fat dry milk for 1 hour at room temperature. After blocking, membranes were probed with the indicated primary antibody overnight at 4 °C and then blotted with the respective secondary antibodies. The membranes were analysed by the use of super ECL detection reagent (Applygen, Beijing, China).

The following antibodies were used: anti-PLK1(dilution 1:1,000, Upstate), anti-Ki67 (dilution 1:500, Abcam), anti-caspase3 (dilution 1:500, Proteintech), anti-Myc (dilution 1:500, Santa Cruz Biotechnology), anti-ERK1/2 (dilution 1:500, Cell Signaling Technology) and anti-β-actin (dilution 1:5,000, Sigma).

### Statistical Analysis

Data are expressed as the mean ± SD from 3 independent experiments. Comparisons of continuous variables between groups were conducted using the Student’s t-test or one-way analysis of variance. PRISM 5.0 (GraphPad Software, Inc., San Diego, CA) was used to perform the data analysis. *P* values < 0.05 were considered statistically significant.

## Results

### Sepsis-induced intestinal barrier dysfunction

**Figure 1:**
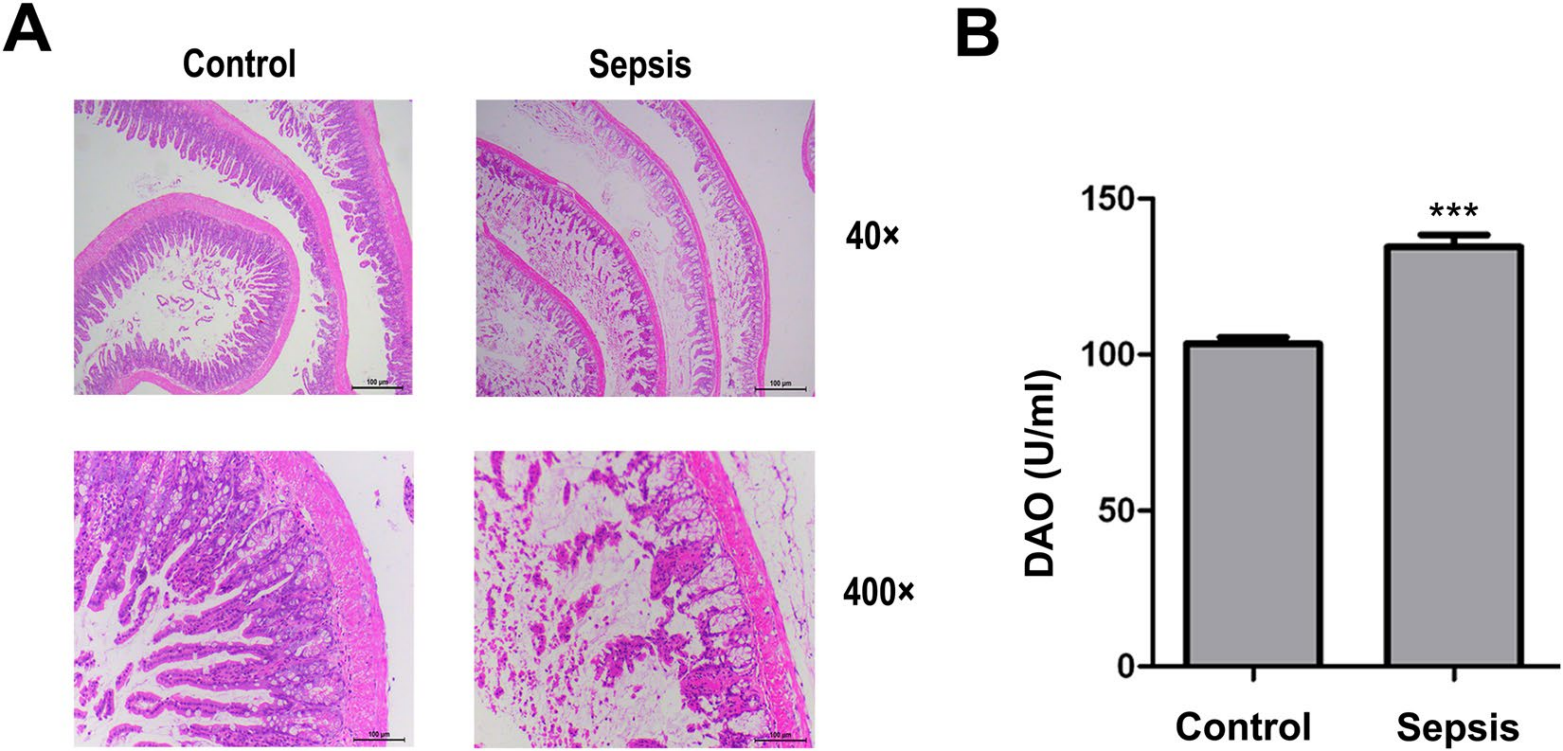
Sepsis-induced intestinal barrier dysfunction. (**A**) Intestinal tissue from the control and sepsis groups at 12 h after injection of LPS (haematoxylin and eosin staining); sections are representative of ten animals in each group. (**B**) DAO (a marker of intestinal permeability) concentrations in serum 12 h after administration of LPS. DAO concentrations are expressed as the mean ± SD. ***P < 0.001, control versus sepsis (n = 10).

To evaluate the intestinal barrier function during sepsis, we examined H&E-stained sections of the intestinal mucosa and measured DAO concentrations to assess intestinal permeability. In animals of the sepsis group, the intestinal mucosa appeared atrophic, with loss of intestinal villi (Fig. 1A), and the serum DAO concentration was increased (Fig. 1B).

### Sepsis suppresses intestinal epithelial cell proliferation and induces apoptosis

**Figure 2:**
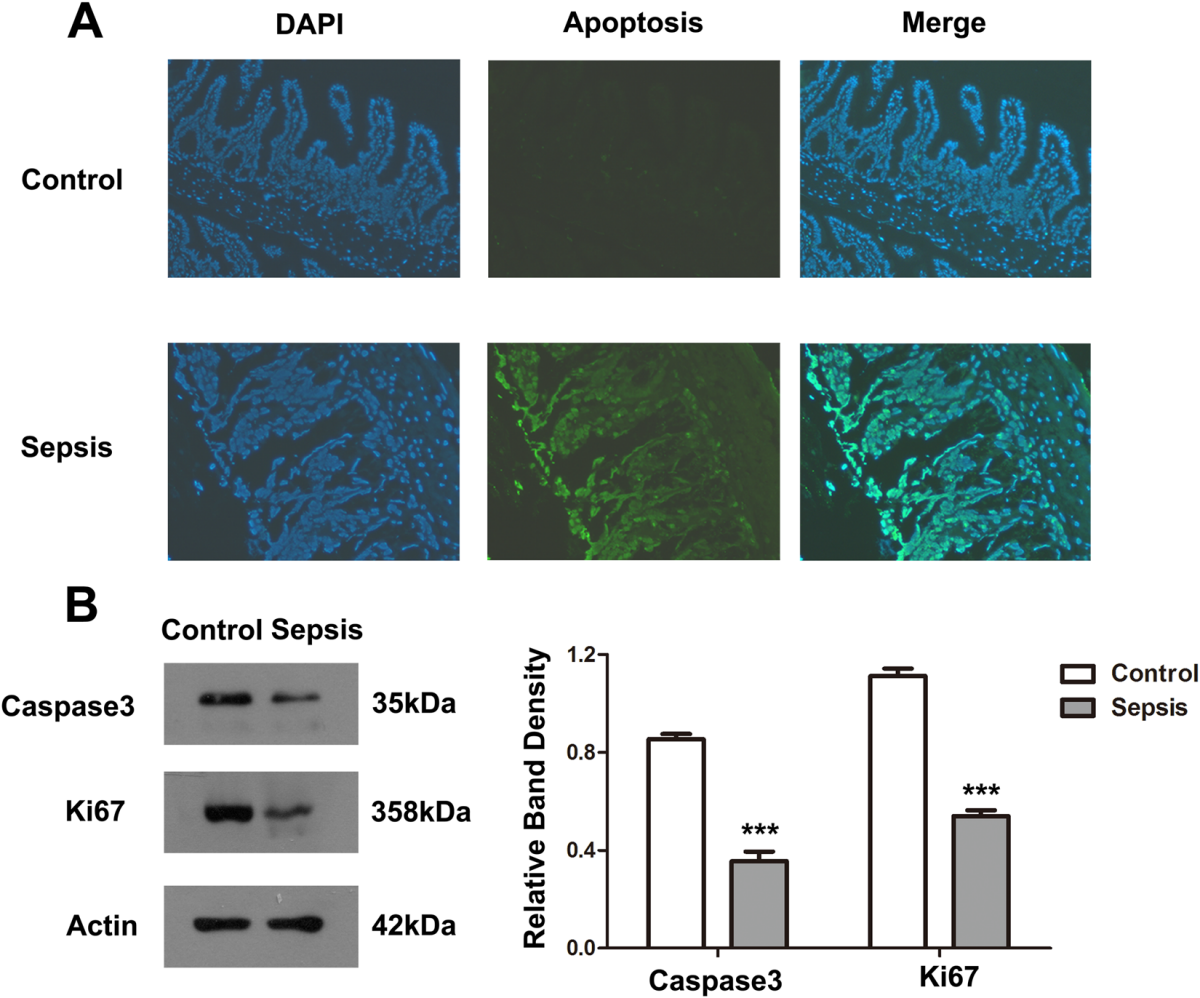
Sepsis suppresses intestinal epithelial-cell proliferation and induces apoptosis. (**A**) TUNEL assay was used to determine apoptosis in intestinal epithelium. The green-stained (fluorescein isothiocyanate (FITC)) cells are apoptotic cells. (**B**) Intestinal tissue protein of each group was extracted, and western blot was used to determine the levels of Ki67 and caspase3. Actin was used as control. The graph represents the relative band densities. Values are mean ± SEM (n = 3). ***P < 0.001 versus control group.

We hypothesized that the disequilibrium between proliferation and apoptosis in intestinal epithelial cells would result in intestinal barrier dysfunction. To test this, we conducted TUNEL and western blot experiments on intestinal epithelium. We found the number of apoptotic cells in intestinal epithelium of the sepsis group was markedly greater than in the control group (Fig. 2A). The levels of caspase3 and Ki67 were significantly decreased in the sepsis group (Fig. 2B).

### PLK1 down-regulation in intestinal epithelium during sepsis

**Figure 3:**
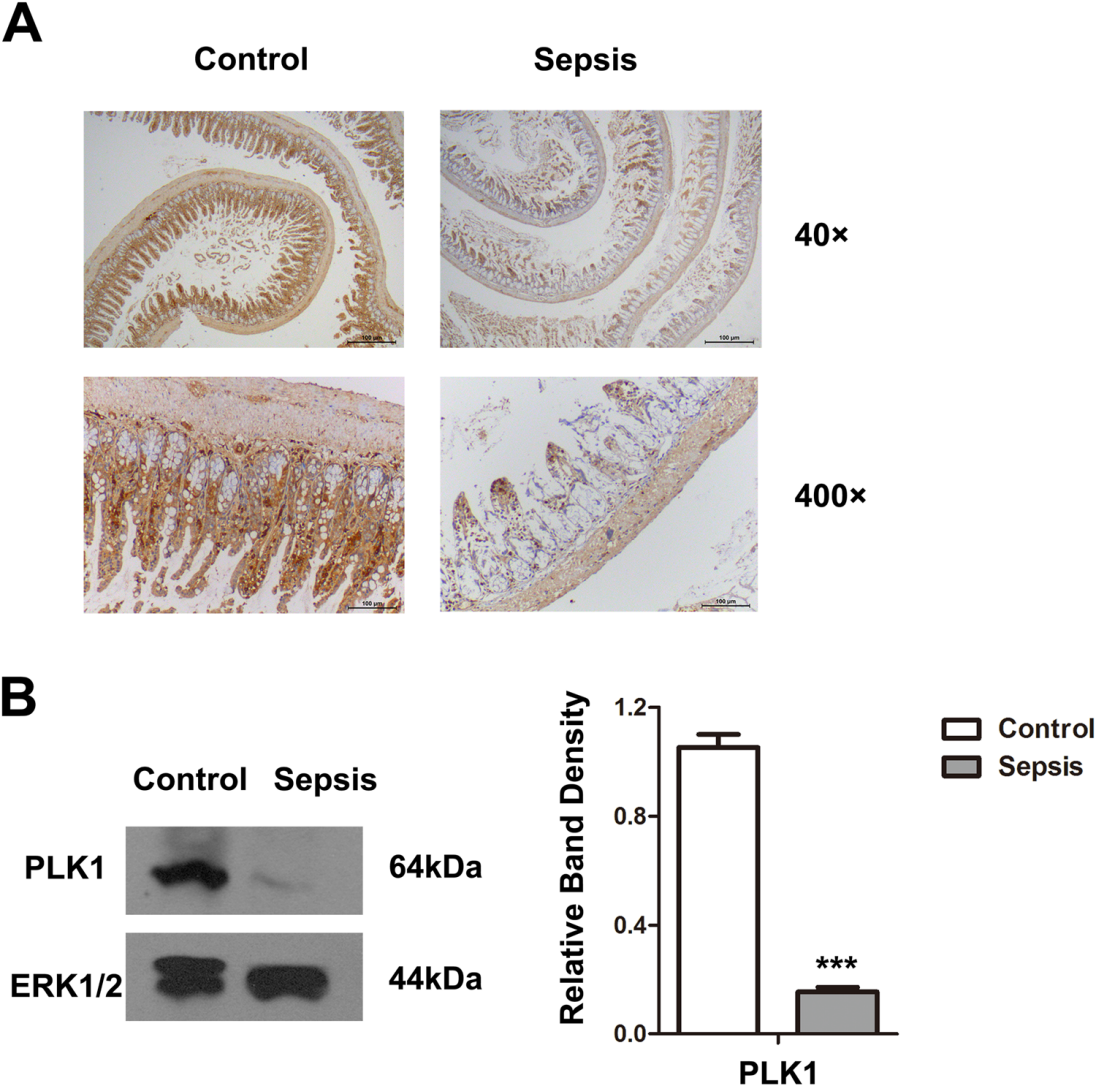
Sepsis induces PLK1 down-regulation in intestinal epithelium. (**A**) Immunohistochemical staining for PLK1 in intestinal tissue sections of control and septic animals. (**B**) Western blot analysis of PLK1 in intestinal tissue of control and septic mice. ERK1/2 was used as control. The graph represents the relative band densities. Values are mean ± SEM (n = 3). ***P 0.001 versus control group.

PLK1 overexpression contributes to resistance to apoptosis, and knockdown of PLK1 leads to apoptosis in oesophageal squamous cell carcinoma cells^13,14^^.^ To determine whether PLK1 is involved in sepsis-induced apoptosis of intestinal epithelial cells, we examined the expression of PLK1 with immunohistochemistry and western blot. PLK1 expression was decreased in intestinal tissue in septic mice (Fig. 3A,B).

### LPS suppresses proliferation in HT29 Cells

**Figure 4:**
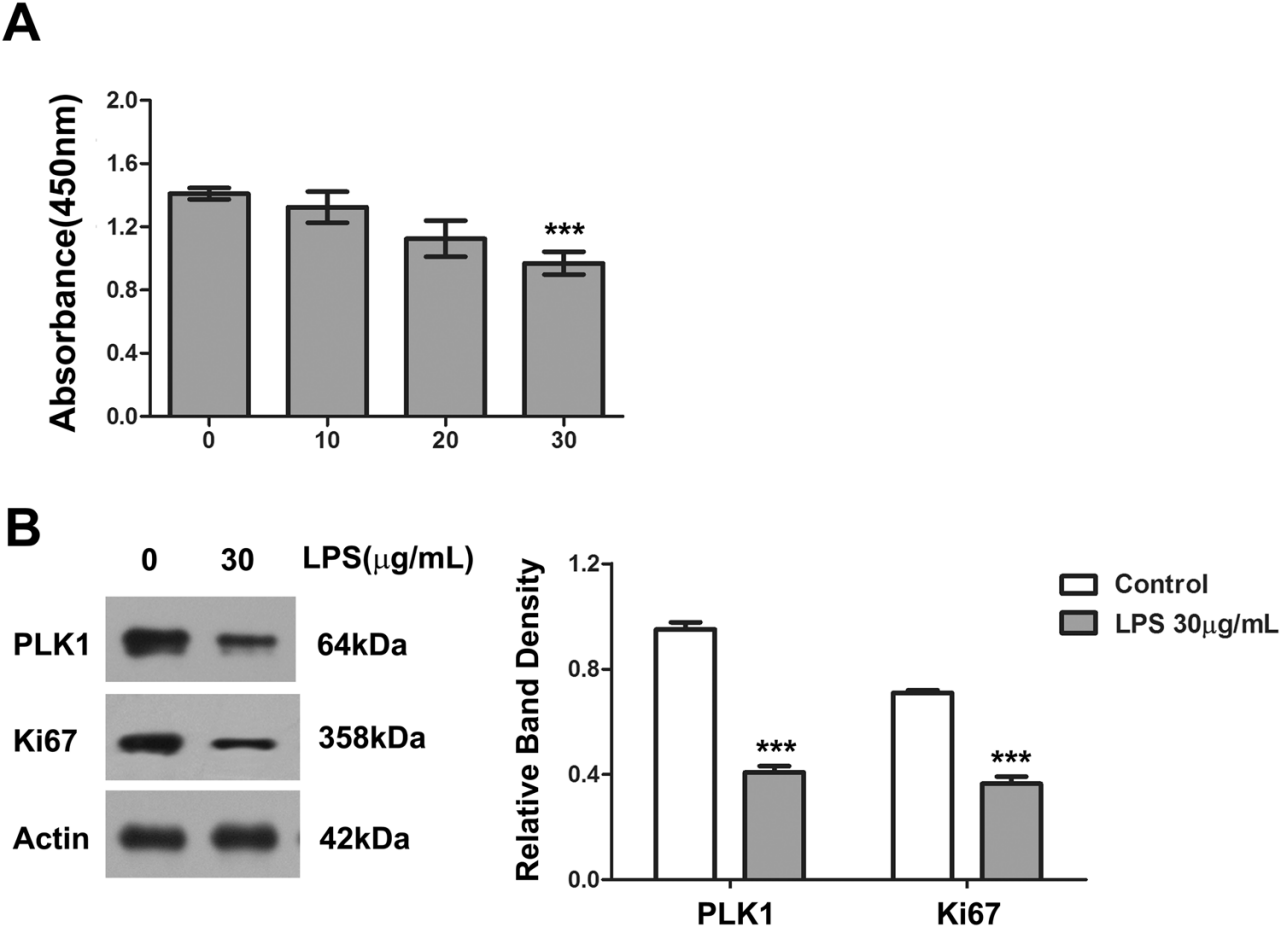
LPS suppresses proliferation in HT29 Cells. (**A**) The cell-proliferative inhibition effect of LPS in HT29 cells (CCK-8 assay). ***P < 0.001 compared with control group. (**B**) The levels of PLK1 and Ki67 in HT29 cells after treatment with 30 μg/mL LPS for 24 h. The graph represents the relative band densities. Values are mean ± SEM (n = 3). ***P < 0.001 versus control group.

To explore the underlying mechanism of intestinal barrier dysfunction in sepsis, we first examined the effect of LPS on cell proliferation in HT29 cells. Exposure to LPS for 24 h inhibited the growth of HT29 cells in a dose-dependent manner (Fig. 4A). We then tested the expression of Ki67, a marker of proliferation, in LPS-treated HT29 cells. The expression of Ki67 was markedly decreased after treatment with LPS (30 μg/ml) for 24 h (Fig. 4B).

### LPS induces apoptosis in HT29 cells

**Figure 5:**
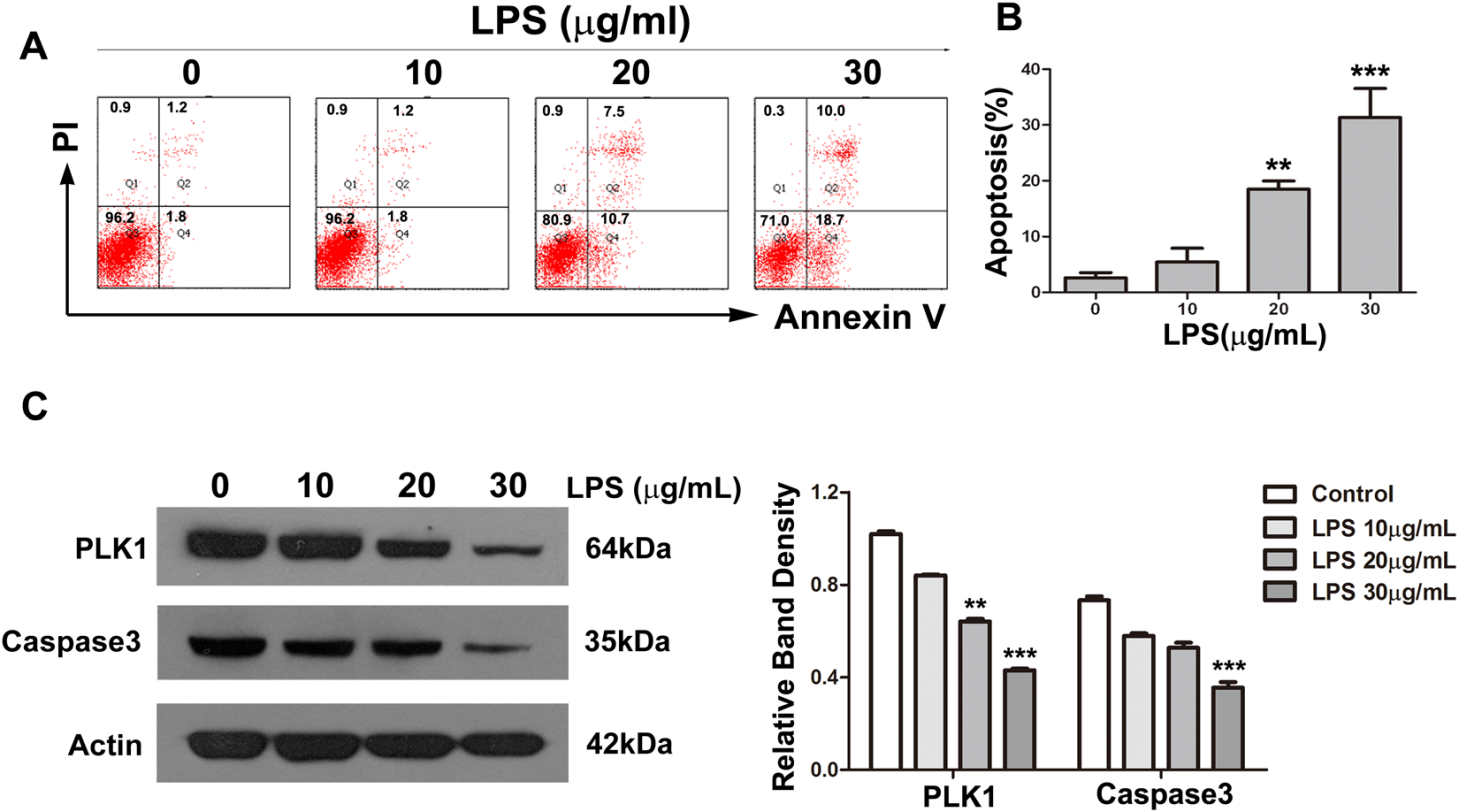
LPS induces apoptosis in HT29 cells. (**A**) HT29 cells were exposed to various concentrations of LPS for 24 h. Apoptosis was analysed by Annexin V-FITC/PI double-labelling assay. (**B**) The degree of apoptotic cell death was quantified. Data represent the mean ± SD (**P < 0.01, ***P < 0.001 compared with control group.). (**C**) The levels of PLK1 and caspase-3 in HT29 cells after treatment with LPS at various concentrations for 24 h. The graph represents the relative band densities. Values are mean ± SEM (n = 3). **P < 0.01, ***P < 0.001 versus control group.

We used Annexin V-FITC/PI double-labelled flow cytometry analysis for the detection of apoptotic cells. The proportion of apoptotic cells was less than 10% in HT29 cells treated for 24 h with control vehicle only (0.9% NS). In contrast, the proportions of apoptotic cells (total of early + late apoptosis) after treatment with LPS at 10, 20 and 30 μg/ml were 5.4%, 18.5% and 31.3%, respectively (Fig. 5A,B). With increasing doses of LPS, the level of caspase3 gradually decreased (Fig. 5C).

### LPS down-regulates PLK1 in HT29 cells

According to the results of immunohistochemistry and western blot, PLK1 was significantly reduced in intestinal tissue of septic mice. To further examine the relationship between PLK1 and sepsis, we treated HT29 with various doses of LPS and then detected the expression of PLK1. With increasing doses of LPS, PLK1 gradually decreased (Figs 4B and 5C).

### Over-expression of PLK1 partly rescues the apoptosis and proliferation inhibition caused by LPS in HT29 cells

**Figure 6:**
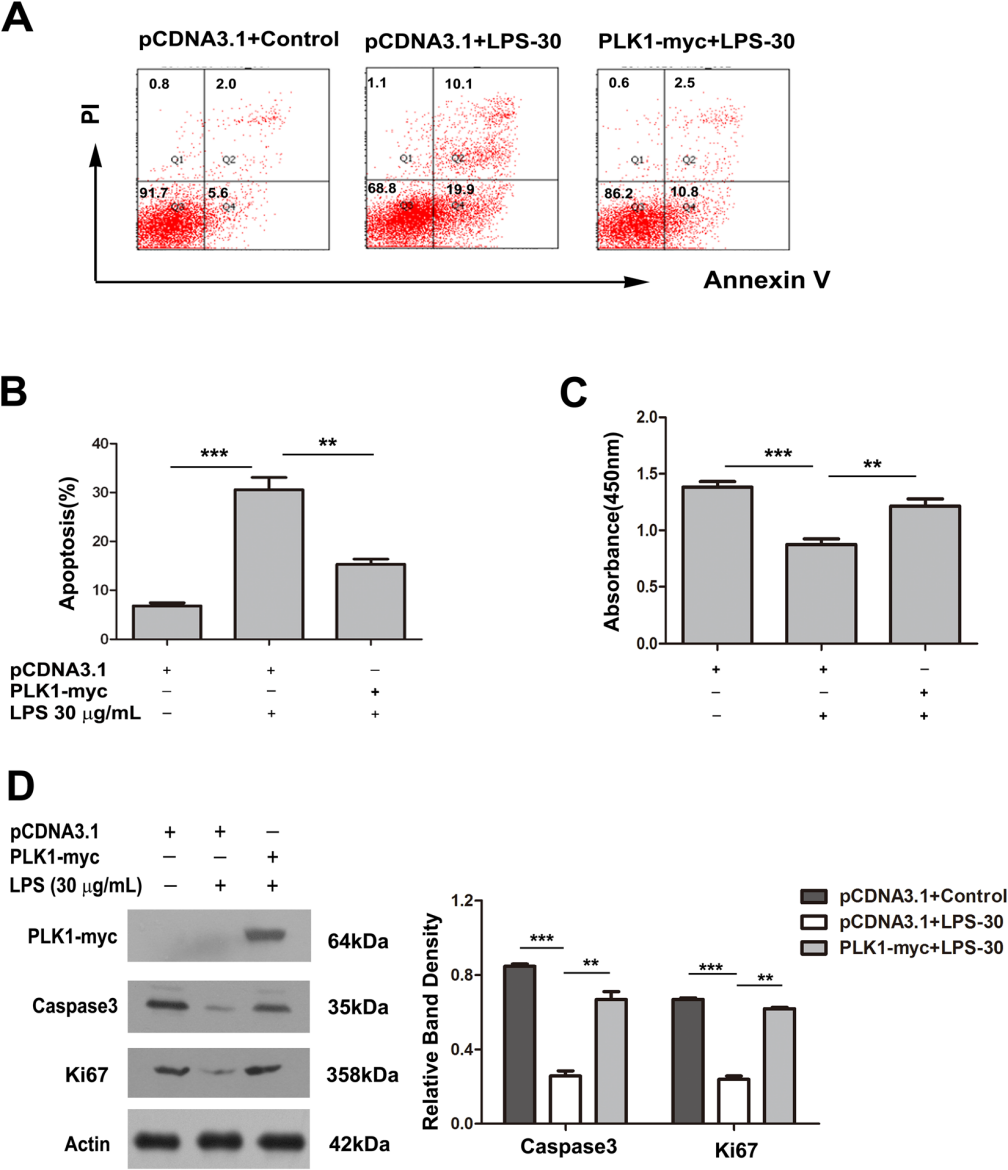
Over-expression of PLK1 partly rescues the apoptosis and proliferation inhibition caused by LPS in HT29 cells HT29 cells were transfected with pcDNA-PLK1-myc or the control cDNA for 24 h, then exposed to LPS (30 μg/ml) for 24 h. (**A**) A representative result of apoptosis analysed by Annexin V-FITC/PI double-labelling assay in HT29 cells after the above treatments. (**B**) Percentages of apoptotic cells in HT29 cells after the above treatments. Values are mean ± SEM (n = 3). **P < 0.01, ***P < 0.001. (**C**) The absorbance at 450 nm of HT29 cells after the above treatments. Values are mean ± SEM (n = 3). **P < 0.01, ***P < 0.001. (**D**) The levels of Ki67 and caspase3 after the above treatments. The graph represents the relative band densities. Values are mean ± SEM (n = 3). ***P < 0.001 versus control group. **P < 0.01 versus LPS group.

To determine whether the lack of PLK1 contributed to the LPS-induced apoptosis and proliferation inhibition in HT29 cells, PLK1-overexpressing HT29 cells were exposed to 30 μg/ml LPS for 24 h, then the proliferation and apoptosis of cells were assessed. LPS-induced apoptosis and proliferation inhibition were significantly prevented by PLK1 cDNA transfection in HT29 cells (Fig. 6A–D).

## Discussion

In this study, we found that proliferation and apoptosis of intestinal epithelial cells play critical roles in sepsis-induced intestinal mucosal barrier dysfunction, and the down-regulation of PLK1 is involved in the intestinal epithelial proliferation inhibition and apoptosis that occurs with sepsis.

The main component of the intestinal barrier is the epithelial cells of the mucosa^15,16^. Apoptosis of the epithelial cells destroys the tight junctions between cells and thereby increases intestinal permeability^17^. Whether intestinal mucosal barrier dysfunction is a consequence of inflammatory response or a primary cause of mucosal inflammation is still unclear^18^. Other studies^19–21^ have demonstrated that proinflammatory cytokines disrupt intestinal barrier function both *in vitro* and *in vivo*, observations that are consistent with our finding that, in septic mice, the intestinal mucosa was damaged, with hyperaemia and oedema. Restoration of intestinal barrier function is a meaningful therapeutic strategy in sepsis.

The integrity of the intestinal mucosal barrier depends on the balance of epithelial cell proliferation and apoptosis^22,23^. PLK1, as a member of the polo-like kinase family, which are highly conserved serine/threonine kinases, plays critical roles in centrosomes at the G2/M transition, separation of sister chromatids, assembly of mitotic spindles, and cytokinesis^24^. PLK1 usually is highly expressed in embryonic tissues, corresponding to embryonic cells’ high proliferation rate. In the adult, PLK1 can be detected in proliferative tissues, such as bone marrow and epithelium, indicating that PLK1 expression has a bearing on cell proliferation^25–27^. Overexpression of PLK1 promotes cell proliferation, and depletion of PLK1 results in an inhibition of proliferation and induces apoptosis in other tissues^28–30^. In this study, we found that PLK1 was down-regulated during sepsis *in vivo* and vitro, and we propose that the down-regulation of PLK1 disrupts the balance between proliferation and apoptosis of intestinal epithelial cells in sepsis.

We acknowledge that our study has some limitations. First, we used intraperitoneally injected LPS to establish the sepsis model, which doubtless is not representative of the various types of sepsis encountered in clinical settings. Second, we were not able to use PLK1^+/+^ mice; instead we over-expressed the PLK1 gene in HT29 cells as a means of testing the role of PLK1 in sepsis.

In conclusion, this study has contributed to understanding the mechanisms involved in the disruption of the intestinal mucosal barrier in sepsis. The results indicate that sepsis-induced intestinal barrier dysfunction may be the result of disequilibrium between proliferation and apoptosis in intestinal epithelial cells, which is caused by the down-regulation of PLK1. These observations might be useful in the development of measures to treat sepsis-induced intestinal barrier dysfunction.
